# “I’ve kept going” – a multisite repeated cross-sectional study of healthcare workers’ pride in personal performance during the COVID-19 pandemic

**DOI:** 10.1186/s12913-023-09246-5

**Published:** 2023-03-31

**Authors:** Kristina Bondjers, Ingebjørg Lingaas, Synne Stensland, Dan Atar, John-Anker Zwart, Hilde Wøien, Grete Dyb

**Affiliations:** 1 Norwegian Centre for Violence and Traumatic Stress Studies, Oslo, Norway; 2 Research and Communication Unit for Musculoskeletal Health (FORMI), Oslo University Hospital, Oslo, Norway; 3 Department of Cardiology, Oslo University Hospital Ulleval, Oslo, Norway; 4 Institute of Clinical Medicine, University of Oslo, Oslo, Norway; 5 Department of Research and Innovation, Division of Clinical Neuroscience, Oslo University Hospital, Oslo, Norway; 6 Division of Emergencies and Critical Care, Oslo University Hospital, Oslo, Norway

**Keywords:** COVID-19, Health professionals, Occupational health, Work environment, Pandemics

## Abstract

**Background:**

For healthcare workers, working through a pandemic may include both challenges, such as coping with increased demands and a lack of control, and rewards, such as experiencing a sense of achievement and meaningfulness. In this study, we explore the accomplishments healthcare workers themselves are proud of achieving at work, in order to elucidate the positive aspects of working through a pandemic.

**Methods:**

In June 2020 (T1), December 2020 (T2), and May 2021 (T3), healthcare workers (n = 1,996) at four Norwegian hospitals participated in a web-based survey assessing job strain, psychological health, and support during the pandemic. The survey included the open-ended question *“During the past two weeks, what have you been feeling proud of achieving at work?”*. Responses (1,046) to this item were analyzed using conventional content analysis, which resulted in 13 subthemes under 6 themes.

**Results:**

For some, pride was found in their professional identity and dedication to their work. Others took pride in specific achievements, such as juggling their own needs (e.g., health, private life) with those of the workplace, contributing to cohesion and collaboration, their ability to learn and adjust, in being a useful resource at work, and in their efforts towards developing the organization and workplace.

**Implications:**

The current findings shed light on what healthcare workers feel proud of achieving in their day-to-day work. Assessment of these factors provides insight on both positive and negative aspects of working clinically during a pandemic, and highlights specific targets for building sustainable and rewarding work environments for healthcare workers.

## Introduction

Healthcare workers are a vital part of the societal response to pandemics and large-scale medical emergencies. During the Coronavirus Disease 2019 (Covid-19) pandemic they continued to work through multiple outbreaks, with varying access to medical, physical, and human resources. The risk of infection, quarantining, and transmitting disease to colleagues or vulnerable patients, along with serious capacity problems (e.g., staff shortages, lack of hospital beds and medical equipment), have all been potential strains throughout the pandemic [1, 2]. Capacity problems may lead to increased workload, a need to swiftly reallocate personnel to other units, and the necessity of enforcing reprioritizations of planned procedures and ordinary healthcare [3–7]. The challenges faced by healthcare workers extend beyond the workplace, and include fear of spreading disease to family or friends and difficulties maintaining a healthy work-life balance in a situation where work demands a lot of time [8–10].

Studies examining the experiences of frontline workers during the Covid-19 pandemic have found that while workers have experienced difficulties such as those described above, there are also unexpected positives. Such positives include feeling that their work is meaningful, experiencing closer interpersonal relationships at work and in their private lives, shifts in values and priorities, seeing the community come together, increased density of staff, feeling dedicated, and feeling proud of their work [3, 11–16]. Pride is felt in response to achievements attributed to one’s own performance and is closely related to feelings of joy and meaningfulness [17, 18]. In turn, this sense of pride affects work satisfaction, performance, perseverance, and turnover intention [19–23]. While some studies report that healthcare workers have taken pride in their work during the pandemic, they tell us little about which achievements and experiences elicit this pride. To recognize healthcare workers’ efforts, we must first identify the achievements they regard as especially valuable. Such knowledge could provide important information not only on what has been perceived as challenging, but also on how to reinforce positive workplace behaviors and improve performance and job satisfaction [24, 25].

This study aims to add to current knowledge of positive and challenging aspects of healthcare workers’ experiences during a pandemic, by examining sources of provide in personal achievements and performance.

## Methods

### Procedure and data collection

This study applied data from an ongoing longitudinal open-cohort study following hospital personnel in Norway during the Covid-19 pandemic. The study received ethical approval from the Norwegian Ethical Review Authority (ref no 130944). Informed consent was obtained from all subjects. We aimed to collect data during infection peaks to capture the most stressful time periods during the outbreak. Participants were working at four large university hospitals (Oslo University Hospital, Akershus University Hospital, St Olavs Hospital, and University Hospital of North Norway). In April/June 2020 (T1), December 2020 (T2), and April 2021 (T3), invitations to participate in a web-based survey were sent out to hospital personnel using the hospitals’ normal channels for communicating with their staff (e.g., e-mail, SMS, online bulletin boards). The data in the current study comprised free-text responses to an open-ended question: *“During the past two weeks, what have you been feeling proud of achieving at work?”*

### Setting

In Norway, the government declared the pandemic a national crisis on March 12th 2020 [26]. The first wave of rapidly increasing Covid-19 cases peaked in April–May 2020, the second wave in December 2020–January 2021, and the third wave in May 2021. As of today (June 1st 2022) there have been 1,433,260 reported cases, 3,141 deaths, and 12,514 admissions to hospital, including 1,979 admissions to intensive care units [27]. While the rate of infection has been lower in Norway compared to other European countries, Norwegian hospital wards have made considerable organizational changes including introducing infection control measures, cancelling elective care, implementing new procedures, and reassigning personnel to new wards to meet demands related to an increased influx of patients with Covid-19 [28, 29].

### Participants

In total, 1,996 participants took part in the study at T1–T3, with 1,046 participants providing a response to the open-ended question about work-related pride (T1: 481, T2: 486, T3: 279). Most participants were female (76%, n = 793). Table 1 provides demographic details about age, gender, profession, and work with patients with COVID-19 at all three timepoints.

**Table 1:** Demographic characteristics

	T1 (n = 481)	T2 (n = 486)	T3 (n = 279)
Age (years, M (SD))	43.5 (11)	42.4 (12)	45.0 (11)
Sex (Female) (n, %)	384 (80)	370 (76)	203 (73)
Professional role			
Nurse	288 (60)	217 (45)	129 (46)
Physician	90 (19)	81 (17)	52 (19)
Other clinical	66 (14)	130 (27)	68 (24)
Non-clinical	37 (8)	57 (12)	30 (11)
Working with patients with COVID-19			
No	137 (29)	209 (43)	125 (45)
Yes, but not severely ill	147 (33)	160 (33)	65 (23)
Yes, severely ill	167 (37)	115 (24)	89 (32)

M = mean, (SD) = standard deviation

### Data analysis

We employed conventional content analysis, following the procedure suggested by Hsieh and Shannon (2005) [30], to compile 1,246 responses into themes. Two authors (KB, IL) began by reading and discussing the contents of 100 free-text responses and possible codes. These results were discussed with one other author (SS), and then used as a guide for the subsequent coding of remaining responses performed by authors KB and IL. The authors coded half of the responses each, and discussed their coding throughout the process in order to maintain the same level of content and prevent any drift in the coding strategy. Each free-text response resulted in between 1 and 7 codes. Next, codes were categorized based on similarity of content, and sorted into themes and subthemes. The themes were discussed by authors KB, IL, GD, HW, and SS. When a proposed model was agreed upon, the remaining authors gave their input. The analysis was finalized when all the authors agreed upon the structure and theme names and when illustrative quotes had been selected. Selected quotes were translated into English by a native English speaker.

## Results

**Fig. 1:**
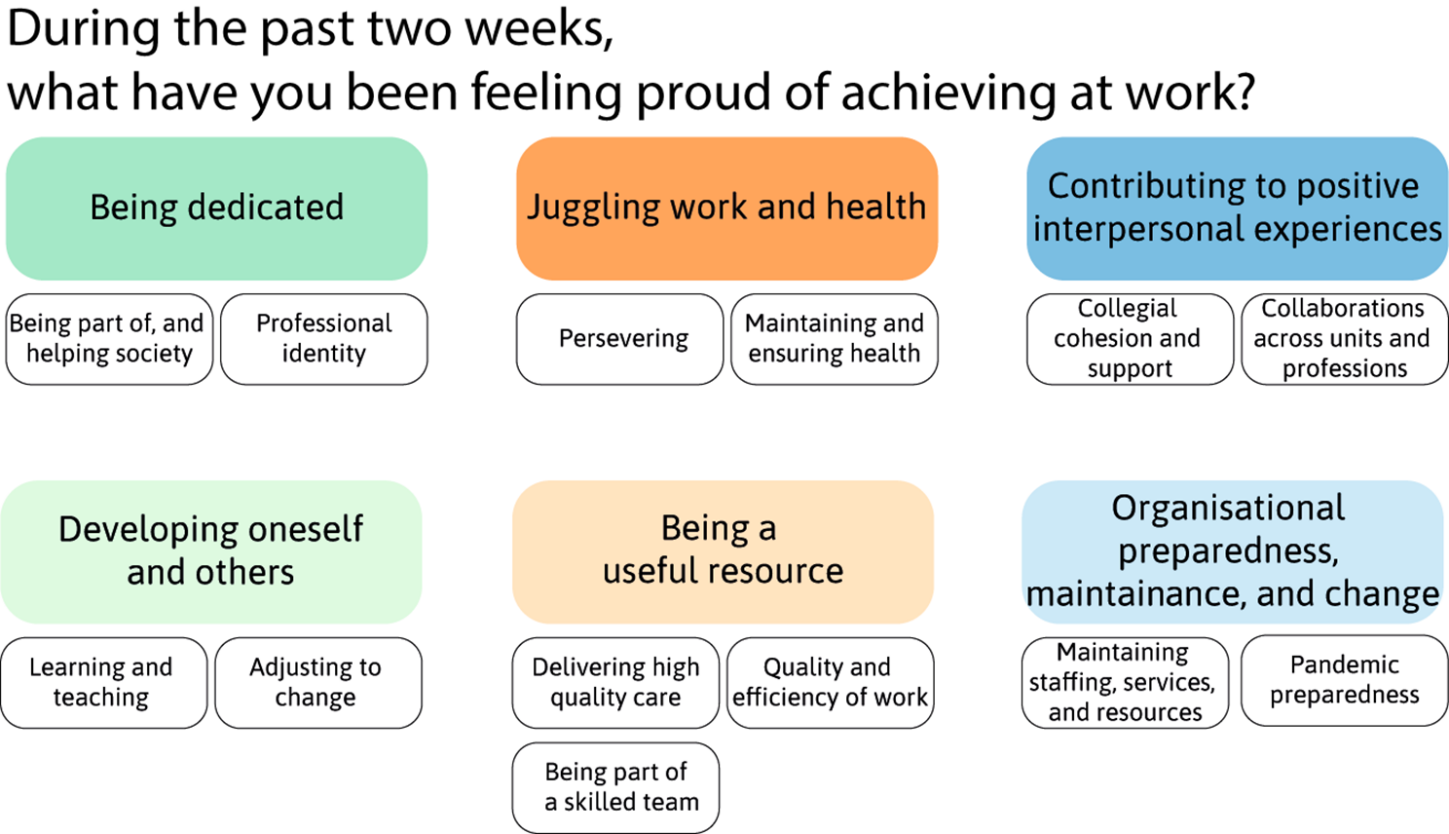
Graphic representation of model

The analysis resulted in 6 main themes describing sources of pride in personal performance, each with 2–3 subthemes (presented graphically in Fig. 1).

### Being dedicated

This theme included the subthemes: ‘Being part of and helping society’ and ‘Professional identity’. Participants found satisfaction in being part of a community fighting the pandemic and in being a resource to society. They also expressed finding gratification in their work, and felt proud of having chosen an important profession and dedicating themselves to their work. Some said it was a privilege to work closely with people in vulnerable circumstances.

I really love my job. It is fantastic to be where things are happening. I don’t think of myself as a “helper”. I am privileged to be so close to another person on existential topics like life and death. Hopefully there can be a light in the darkness. And I am so privileged to share a small part of the journey. (T2)

### Juggling work and health

The theme includes the subthemes: ‘Maintaining and ensuring own health’, and ‘Persevering’. Participants were proud of their ability to stay calm and in good spirits. Avoiding disease and distress, such as infection, burnout, or feeling overwhelmed, recovering from sickness, and making changes in their work situation (e.g., finding new positions or assignments) in order to keep working were mentioned as sources of pride. Maintaining an adequate work-life balance, and, in some instances, going on sick leave, inspired pride, as these steps were vital for protecting personal health and patient safety.

I managed to ask my GP for a sick note because I am burned out, the patients are spared having me distracted at work. (T3)

Persevering throughout the pandemic could simply mean continuing to go to work without experiencing enduring negative emotions.

That I always manage to come to work with the intention of contributing to making a difference for every patient and co-worker. (T2)

There were differences between timepoints within this theme. At the first assessment point (T1) some participants stated that they were proud of surviving. This code did not emerge at the later timepoints.

### Contributing to positive interpersonal experiences

The theme included the subthemes ‘Collegial cohesion and support’ and ‘Collaborations across units and professions’. Participants were proud of spreading positivity among colleagues by, for example, cheering them on, improving the physical and social work environment, bringing cake to work, or contributing to an overall good cohesion and mood. Taking care of especially vulnerable colleagues, such as those re-assigned to new work tasks or with temporary positions, was mentioned.

Supported many nurses that have now been forced to relocate to us. Helped them to experience it positively even though they didn’t want to come to us. (T1)

From a management perspective, following up with employees with both emotional and practical support was described as a source for pride. Working and collaborating across professions, such as pre-hospital and hospital personnel, or between nurses and physicians, and units/clinics, was mentioned as a source of pride. Some mentioned the specific outcomes of a successful collaboration, such as enabling proper care for patients.

### Developing oneself and others

The theme included the subthemes ‘Adjusting to change’ and ‘Learning and teaching’. Participants were proud of being able to swiftly adapt to new circumstances such as working with new colleagues, new procedures, and with patients suffering from an unknown disease. Some participants mentioned a lack of support or training.

*Juggle a work day in completely unknown settings over and over again where we don’t know what the emergency preparedness is. Am also proud of how we handle switching between covid wards and regular intensive care, and not knowing the people you are working with. (T2)*

Participants expressed pride in improving their own competence, both formally in terms of promotions or advancements, but also informally by learning new tasks or procedures, staying updated on current research, and in training others. Mastering tasks that had not been performed for a long time was seen as an achievement.

Was responsible for an intensive care patient with minimal training and X years since I worked at the intensive care unit (T1)

As for training others, participants expressed pride in the learner’s progress, and said it felt important to share their knowledge with others even though it could be time-consuming.

### Contributing as a useful and vital resource

The theme included the subthemes ‘Delivering high quality care for patients and relatives’, ‘Being part of a skilled team’ and ‘Maintaining or increasing work efficiency during trying times’. High quality care included successful medical outcomes for patients (e.g., stabilizing severely ill patients, successful procedures, improvements, discharge from intensive care units), successful rehabilitation, making decisions about patient prioritization, providing emotional support and a feeling of safety, and caring for patients’ relatives.

Prioritizing certain patients, for example deciding who to meet physically or digitally in open-care settings, and being able to focus on their patients’ needs was also mentioned as a source of pride. Participants described managing difficult clinical situations, such as providing comfort despite protective gear creating a barrier or providing dignified palliative care.

*That relatives were able to give a dignified farewell to a dying cancer patient despite all the Covid restrictions. (T2)*

Being part of a skilled team that was able to adjust and manage stressful clinical situations and being proud of one’s colleagues were also sources of pride. Teams were described as knowledgeable, flexible, and competent.

We have a lot of professional competence in the emergency room, and I am proud of that! And we all take the challenge head on despite having few resources. (T2)

Maintaining efficiency was mentioned as a source of pride. Staying on top of specific tasks such as lab work, research, and administrative work were described as important. Keeping up with an overall heavy workload, taking on extra shifts, and multitasking were also mentioned. Some participants said that their teams’ work efficiency increased during the pandemic, and others that they were proud to maintain efficiency despite scarce resources and little support.

Managed to complete almost all our tasks despite having insufficient staff. (T1)

### Organizational preparedness, maintenance and change

This theme included the subthemes ‘Pandemic preparedness’, and ‘Ensuring and maintaining proper staffing, resources, and services’. This entailed reorganizing units to covid wards, revising pandemic procedures to ensure that guidelines were up to date, ensuring access to protective gear, implementing infection measures, and, at T2 and T3, organizing and prioritizing vaccinations. Both establishing and complying with routines were highlighted as important.

That we enforcing physical distancing and spread out in smaller seating areas including in the hallways, and use facemasks as needed when we are in close contact with our co-workers. In this way we have prevented contagion many times when we have had sick colleagues at work. (T2)

Ensuring and maintaining proper staffing, resources, and services by utilizing all available supplies, reorganization of unutilized resources and staff, hiring new staff, and keeping staff healthy was mentioned as a source of pride. Some mentioned the units’ or hospitals’ ability to continue to provide ordinary healthcare.

That we have given the cardiac patients the care they need throughout the pandemic and that we have kept the staff healthy. (T3)

Standing up for their own rights and safety as well as for their colleagues as a union representative was mentioned by some participants:

*I am a union representative. I am actually proud that we sent in a message of concern on our union’s behalf because of the workload we have been under. (T3)*

### Pride despite of hardships

In some instances, respondents mentioned experiencing specific challenges (i.e., personal, collegial, or organizational) alongside their achievements. These included feeling proud of completing their duties and contributing at work despite a lack of staff, protective gear, or personal motivation, dissatisfaction with leadership, or missing reallocated colleagues. One participant mentioned that even though they felt like an important resource within society, they did not feel adequately appreciated or rewarded.

I consider myself to be a good resource in the work we do in a difficult societal situation and often feel that we are not valued for the work we do. Applause is nice but it doesn’t pay the bills. (T2)

For some participants, continuing to work was associated with high personal costs:

I have gotten to work and managed to go all day without crying. Even though I’m completely exhausted and have met the wall. You cry when you are home alone.

### No sense of pride or achievement

Finally, a few participants explicitly stated that they did not feel proud of anything in their performance at work during the past week. Some simple stated *“I do not feel proud”* or *“nothing in particular”*, whereas others mentioned feeling like they did not perform well: *“I’m not proud, I’m not getting any work done”*, or *“I can’t remember the last time I felt proud of anything at work.”*

## Discussion

The purpose of this study was to contribute to our understanding of both positive and challenging aspects of healthcare workers’ experiences during a pandemic by examining sources of pride in personal performance and achievements. Results point out several areas in which healthcare workers take pride. Some took pride in their professional identities and dedication to their work. Others took pride in specific achievements, such as juggling their own needs (e.g., health, private life) with those of the workplace, contributing to cohesion and collaboration, their ability to learn and adjust, in being a useful resource at work, and in their efforts towards developing the organization and workplace. While the majority of participants described sources of pride, a few said that they did not feel any pride.

A noteworthy result from this study is that many aspects of high-quality clinical work elicit pride. Participants described taking pride in both performing and being successful in medical tasks ensuring patient recovery and survival, and in tasks that might be difficult to perform in settings where resources were lacking, such as taking some extra time for patients and their relatives, tending to emotional needs, and providing comfort. Even in situations where a patient did not recover, participants expressed feeling proud of providing compassionate palliative care which allowed for a dignified death and farewell. These findings are in line with previous research [3, 31, 32], and underline that the ability to provide both psychosocial and medical care, tend to every aspect of a patient’s health, and to provide advanced treatments are important aspects in healthcare workers’ assessment of their own performance.

Results also suggest that healthcare workers take pride in juggling their determination to work with their own health needs. This was mentioned in relation to being successful in using coping strategies (e.g., maintaining a positive mindset, leaving work at work) which enabled them to keep working, or in simply persevering (getting to work every day). While some described taking steps to safeguard their own health, such as going on sick leave or shifting positions, others mentioned feeling pride in continuing to work despite the heavy cost to their own psychological or physical health. The difficult balancing act between determination to work and maintaining one’s own health has been described by other authors [11, 32]. This resonates with research suggesting that the pandemic has had a high impact on the health and well-being of healthcare workers [2, 3, 33], and also suggests that healthcare workers are aware of this but take pride in their willingness to put themselves at risk to continue to provide the best possible care, even when the price is high for some individuals.

The importance of maintaining positive collegial relationships, and the active work this requires, are highlighted by this study. Similarly, Kinsella et al. (2021) reported that closer working relationships between colleagues was a benefit of the pandemic. Results from the current study describe concrete measures healthcare workers have taken to improve these relationships, such as taking extra care of reallocated colleagues, bringing cake to work, and contributing to keeping spirits up and inspiring courage among colleagues and staff. Access to social support has previously been suggested as a protective factor against occupational unwellness and turnover intention [34, 35]. Thus, these kinds of acts of collegial support are important for healthcare organizations to reinforce in order to increase organizational and individual resilience and reduce occupational unwellness in times of crisis [36].

Organizational psychology suggests that high levels of job demand do not necessarily have a negative effect on job satisfaction and performance. Rather, they can promote employee growth and development if in occurrence with job control (e.g., self-determination and decision making) and adequate social support [34]. In the current study, participants described their own ability to adjust, develop, learn, and teach, as well as their contribution to organizational adjustment and preparedness. This could be described as individuals taking pride in their efforts to increase control and manage demands, both on an individual and on an organizational level. In addition to describing development of pandemic specific procedures and reorganizations, participants also mentioned organizational tasks that are likely present at other times as well, such as ensuring access to staffing, improving work organization, and taking steps to ensure the safety of colleagues and staff. Interestingly, some participants mentioned becoming more efficient during the pandemic. As described by Byrne et al. (2020), [15] increased medical staffing during the pandemic had positive implications on a range of factors, such as ability to take leave, workplace relations, morale, access to support, and, relating to improved efficiency, quicker clinical decision making.

During the pandemic, healthcare workers have been called heroes, and it is easy to understand the appeal of that narrative. It has, however, been suggested that the act of hero-making may stifle meaningful discussion about the reciprocity that healthcare workers should be able to rely on, in which they care for society and society cares for them.

. These results highlight some instances in which healthcare workers themselves take pride in their work for patients and society, but also suggest that they do not always feel supported. For example, some participants explicitly described not feeling valued by society, or that their performance was independent of, or in spite of, inadequate or inconsistent leadership or organizational culture. While some of these hardships are likely due to difficulties posed by the pandemic which are hard to mitigate (e.g., increased workload, unpredictability), others appear to point out areas for change and preemptive organizational work. For instance, ensuring spaces for personnel to give and receive support, both to one another and from leaders, appears to be important for creating an inclusive workplace.

Data in this study were collected throughout the first three infection peaks, between which patient load, access to resources, and knowledge changed. Some minor differences occurred between timepoints. The codes ‘surviving’ (within the theme ‘Juggling health and work’) and ‘adjusting to home office’ (within ‘Adjusting and developing’) only occurred at the first timepoint (T1). During the third timepoint (T3) (April–May 2021), organization and prioritization of vaccination procedures was mentioned within the theme ‘Organizational preparedness, maintenance, and change’, but not at the previous timepoints.

### Limitations and strengths

This study describes the breadth of healthcare workers’ achievements and performance during the pandemic, rather than their frequency. Therefore, we cannot say which achievements were most prominent, nor whether different activities inspire different degrees of pride. Most participants were females with a nursing profession, and although males and other types of healthcare workers (i.e., physicians, other clinical, and non-clinical) are represented, the results must be interpreted with this in mind. We did not examine possible differences regarding age, gender, or profession. Hence, in future research, possible differences could be examined using a quantitative or a mixed method approach. Strengths of the study include a high level of information power and the use of longitudinal data which has allowed us to explore variances across pandemic timepoints.

## Conclusion

Healthcare workers have taken pride in a wide variety of achievements during the Covid-19 pandemic. These include societal, individual, and organizational contributions, with a focus on caring for the well-being of their patients, colleagues, and staff. While the pandemic has been an extraordinary event, several of the stressors under which healthcare workers have performed are not unique to this setting. Experiencing time pressure, reallocations, difficult prioritizations, heavy workload and having multiple roles is part of working in healthcare. We believe that these results are of interest for healthcare organizations beyond the pandemic, as the challenges they describe are also present in ordinary, day-to-day clinical work. These results highlight some important targets for promoting sustainable and rewarding work environments in which healthcare workers can continue to develop, thrive, and learn, even while under pressure.

## Data Availability

The data that supports the findings of this study are available from the corresponding author upon reasonable request.
